# Fluoroquinolone-resistant *Escherichia coli* carrying *quinolone efflux pump* (*qepA*) with L348–G349 duplication in the absence of V134I substitution

**DOI:** 10.1128/spectrum.00056-26

**Published:** 2026-06-29

**Authors:** Kevin Kariuki, Kirkby D. Tickell, Collins Kigen, James Wachira, Polycarp Mogeni, Doreen Rwigi, Cecilia Mbae, Kelvin Kering, Benson Singa, Judd L. Walson, Samuel Kariuki, Patricia B. Pavlinac, Ferric C. Fang

**Affiliations:** 1 Centre for Microbiology Research, Kenya Medical Research Institute 118982, Nairobi, Kenya; 2 Centre for Clinical Research, Kenya Medical Research Institute 118982, Nairobi, Kenya; 3 Department of Global Health, University of Washington 150331, Seattle, Washington, USA; 4 The Childhood Acute Illness & Nutrition (CHAIN) Network, Nairobi, Kenya; 5 Microbiology Hub, Walter Reed Army Institute of Research—Africa, Kericho, Kenya; 6 Kenya Medical Research Institute, Kericho, Kenya; 7 Department of International Health, Johns Hopkins University, Baltimore, Maryland, USA; 8 Department of Medicine, Johns Hopkins University, Baltimore, Maryland, USA; 9 Department of Pediatrics, Johns Hopkins University, Baltimore, Maryland, USA; 10 Department of Medicine, Division of Allergy and Infectious Diseases, University of Washington 205280, Seattle, Washington, USA; 11 Department of Laboratory Medicine and Pathology, University of Washington 7284, Seattle, Washington, USA; DePauw University, Greencastle, Indiana, USA; Centre d'Infection et d'Immunite de Lille, Lille, France

**Keywords:** quinolone efflux pump, *Escherichia coli*, children, fluoroquinolones, Kenya

## Abstract

We report on five clinical fluoroquinolone-resistant *Escherichia coli* strains carrying a unique variant of the quinolone efflux pump gene (*qepA*). The *E. coli* isolates were recovered from fecal samples of children under 5 years collected at hospital discharge from two referral hospitals in Kenya. Whole-genome sequencing revealed that the isolates belong to sequence types 46 and 354, carrying the *qepA* gene flanked by intI1 integrase and ISCR3 elements. Protein sequence comparison revealed a duplication of the L348–G349 insertion, similar to the *qepA8* variant, but without the V134I substitution. The unique *qepA* variant had 14 transmembrane helices, with the additional LG amino acids located within the periplasmic loop connecting transmembrane helices 9 and 10. Additionally, multiple co-carriage of mutations in *gyrA* and *parC*, as well as antimicrobial resistance determinants to aminoglycosides and beta-lactams, were identified in the isolates. The isolates exhibited phenotypic multidrug resistance and reduced susceptibility to a number of other antibiotics, including ceftriaxone. Additionally, the isolates exhibited an elevated (≥32 µg/mL) minimum inhibitory concentration (MIC) to ciprofloxacin, indicative of high-level resistance. The evolution of *qepA* indicates the persistence and diversification of transferable quinolone resistance determinants conferring low-level resistance, which may be facilitating further selection and dissemination of high-level fluoroquinolone resistance, expanding the mutant selection window in *E. coli* strains found in sub-Saharan Africa (SSA). The identification of a unique *qepA* variant—characterized by a unique L348-G349 duplication and absence of the V134I mutation typical of *qepA8*—demonstrates that the *qepA* locus is continuing to diversify in clinical settings.

**IMPORTANCE:**

Fluoroquinolones remain critical antibiotics for the treatment of bacterial infections in low- and middle-income countries. The increasing prevalence of resistance threatens the effectiveness of these agents. This report describes the detection of a quinolone efflux pump (*qepA*) variant with a unique mutation combination among the *qepA* alleles in *Escherichia coli* clinical isolates from children being discharged from hospitals in Kenya. The plasmid-mediated resistance gene *qepA* contained the insertion of an L348–G349 amino acid duplication without the additional V134I substitution present in the *qepA8* allele. The existence of this *qepA* variant underscores the persistence and ongoing diversification of plasmid-encoded resistance determinants, which may be playing a part in selection for high-level resistance in SSA. This study highlights the need for enhanced genomic surveillance of antimicrobial resistance, antimicrobial stewardship, and implementation of strategies to mitigate the spread of antibiotic resistance.

## OBSERVATION

Increasing fluoroquinolone resistance in Enterobacterales is a significant public health concern due to the limited treatment options for Shigellosis, Salmonellosis, and other Gram-negative bacterial infections in low- and middle-income countries (1). Fluoroquinolone resistance is mediated by quinolone resistance-determining region (QRDR) mutations and plasmid-mediated quinolone resistance determinants (PMQR). The *qepA* PMQR gene confers low-level resistance and alters the mutant prevention concentration to facilitate the secondary acquisition of QRDR mutations that result in high-level increases in fluoroquinolone MICs (2). Thirteen *qepA* alleles (*qepA*1–13) have been identified globally in humans, animals, and the environment (3), including some reports in SSA with detection rates of 8%–12% (4–7) but limited description of the specific variants detected. In Kenya, only *qepA1* and *qepA4* have been reported (8, 9).

Among 188 *E. coli* fluoroquinolone non-susceptible isolates recovered from children enrolled in the Toto Bora trial, 16 (9%) were *qepA*-positive isolates as previously described, with only 0.4% of study participants receiving a fluoroquinolone during their inpatient stay (9). For this study, five of the isolates included in this study were selected from this subset for further characterization based on whole-genome sequencing and variant analysis. The five *E. coli* isolates were recovered from rectal swabs of children under 5 years of age at discharge from two Kenyan referral hospitals in Kisii and Homa Bay counties (0.6773° S, 34.7796° E and 0.6221° S, 34.3310° E) between September 2016 and August 2018 during the Toto Bora trial (10). Children were hospitalized for non-traumatic cases, median age 33.4 months and an average hospital length of 5 days. Fecal samples were collected and streaked directly onto MacConkey agar (Oxoid, United Kingdom) and incubated at 37°C for 24 h. *E. coli* isolates were identified biochemically using an API 20E strip (bioMérieux, Inc., United States) (9, 11). Antimicrobial susceptibility testing was performed by disc diffusion and Etest for ciprofloxacin (bioMérieux, Inc., United States). Zone diameters and MICs were interpreted according to CLSI 2024 guidelines (12). PMQR genes were screened by PCR as previously described (9). Genomic DNA was extracted using the Quick DNA Fungal/Bacterial Miniprep Kit (Zymo Research, Hilden, Germany). Libraries were constructed using an Illumina DNA library prep kit (Invitrogen, CA, USA) for (2 × 150 bp reads) and sequenced in a P1 flow cell on NextSeq 1000 as previously described (13). Quality of the raw reads was assessed with FastQC v0.12.1 (14) (https://github.com/s-andrews/FastQC) and trimmed with fastp v0.20.1 (15). Reads were assembled with Shovill v1.1.0 (https://github.com/tseemann/shovill). Quality of assemblies was assessed using QUAST v5.0.2 (16) and CheckM v.1.2.3 for completeness and contamination. Strain typing was done using MLST v2.23.0 (https://github.com/tseemann/mlst) and pathotypes using ECTyper v2.0.0. Serotype identification was done using EcOH (17) on ABRicate v1.0.1 (https://github.com/tseemann/abricate) and phylogrouping using ezclermont v0.7.0 (18). Core genome multilocus sequence typing (cgMLST) V1 was performed using the *Escherichia coli* scheme implemented in EnteroBase v.1.2.0, and hierarchical clustering (HierCC) V1 was used to assess genetic relatedness among isolates at multiple allelic distance thresholds (19). Acquired antimicrobial resistance genes (ARGs) and mutations that confer resistance were identified using AMRFinderPlus v3.12.8 (20), CARD v1.0.1, and ResFinder (21, 22). Plasmid replicons were identified with https://github.com/tseemann/abricate, using the Plasmidfinder (23). Plasmid reconstruction and typing were done using MobSuite 3.1.9. The *qepA* amino acid sequences were aligned with MEGA v11.0.13 and compared to the protein sequence of *qepA* (WP_012372821.1). NCBI Prokaryotic Genome Annotation Pipeline v.6.10 was used to annotate the genome sequences (24) and visualized using SnapGene v8.0.2 and Easyfig v2.25. To identify the amino acid duplication, other alleles of the *qepA* reference sequences were downloaded from NCBI (3) and aligned in MEGA. Additionally, the *qepA* sequences were compared against reference alleles in CARD using nucleotide and amino acid sequences. Sequence identity was based on the overall similarity of known *qepA* alleles (>99% nucleotide identity), while uniqueness was based on distinct nucleotide or amino acid mutation combinations not reported in curated databases. Transmembrane (TM) domains of *qepA* were predicted using DeepTMHMM v.1.0.24 (https://dtu.biolib.com/DeepTMHMM). Predicted protein structures were generated using ColabFold v1.6.1: AlphaFold2 using MMseqs2 (25) and were aligned in ChimeraX v1.11.1, and structural similarity was assessed using root mean square deviation (RMSD) between aligned residues. All tools were used in default settings.

**Fig 1:**
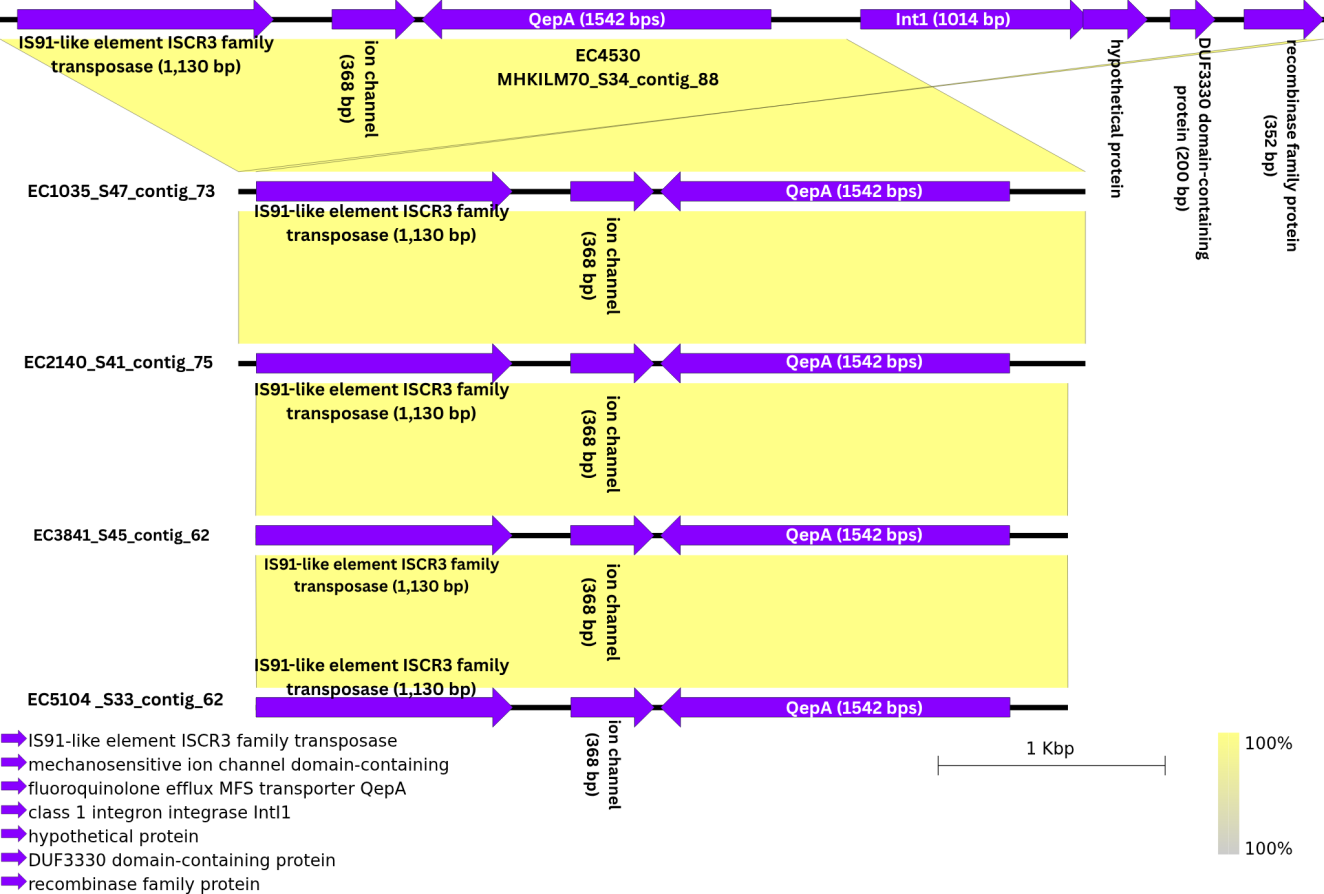
Genetic context of the *qepA* locus in *Escherichia coli* isolates included in this study. Linear comparison of genomic regions surrounding *qepA* across multiple contigs. Arrows indicate open reading frames, with arrowheads showing the direction of transcription. Key genes, including *qepA*, class 1 integron integrase (*intI1*), and IS91-like element (*ISCR3*), are explicitly labeled. All sequences are displayed in the same 5′ to 3′ orientation. Yellow shading between sequences indicates nucleotide identity, with darker yellow shading representing higher similarity. The regions exhibit high sequence conservation (>99% nucleotide identity) with no major structural rearrangements, and no variation between the different isolates is observed in the flanking regions. A scale bar (1 kb) is provided to indicate genomic distance.

Phenotypic characteristics, genome features, sequence types (ST), and phylogroups are provided in Table 1. All isolates had a resistant ciprofloxacin phenotype (MIC ≥ 1 µg/mL) with unique quintuple QRDR mutations (*gyrA*_D87N, *gyrA*_S83L, *parC*_E84G, *parC*_S80I, *parE*_I355T). Other ARGs and mutations associated with resistance to multiple antibiotic classes were also detected in all isolates. Four of the isolates were assigned to ST354 (serotype O153:H34; phylogroup F), whereas EC4530 belonged to ST46 (serotype O9:H10; phylogroup A). cgMLST analysis revealed that the four isolates belonged to the same core genome sequence type (cgST 93,301), while EC4530 belonged to a distinct cgST (206,229). Despite sharing the same MLST, the isolates were assigned to distinct HC0 clusters, excluding clonality, while clustering at higher HierCC levels (HC20–HC50), indicating a common lineage without evidence of recent transmission. Plasmid replicon types identified include IncFIA, IncFIB, IncFIC, IncI2, IncX1, IncX4, IncY, Col (BS512), and Col (MG828). No relaxase, mating pair formation (MPF) system, or origin of transfer (*oriT*) genes were detected in the *qepA*-carrying plasmid in all five isolates, indicating that the plasmids are non-mobilizable. However, linear comparison showed high genetic context similarity with *qepA* genes flanked by truncated intI1 and ISCR3 sequences characteristic of mobile genetic elements (Fig. 1).

**Table:** **Table 1** Phenotypic and genome characteristics of five *qepA E. coli*-carrying strains isolated in Kenya

Strain	EC2140	EC5104	EC4530	EC3841	EC1035
Site	Homabay county	Kisii county	Kisii county	Kisii county	Homabay county
Phenotypic antibiotic resistance profile	Chloramphenicol (S) Gentamicin (R) Cefoxitin (R) Cefotaxime (R) Ampicillin (R) Azithromycin (S) Ceftazidime (R) Ciprofloxacin (R) Ceftriaxone (R) Imipenem (S)	Chloramphenicol (S) Gentamicin (R) Cefoxitin (R) Cefotaxime (R) Ampicillin (R) Azithromycin (S) Ceftazidime (R) Ciprofloxacin (R) Ceftriaxone (R) Imipenem (S)	Chloramphenicol (S) Gentamicin (R) Cefoxitin (S) Cefotaxime (S) Ampicillin (R) Azithromycin (R) Ceftazidime (S) Ciprofloxacin (R) Ceftriaxone (S) Imipenem (S)	Chloramphenicol (R) Gentamicin (R) Cefoxitin (R) Cefotaxime (R) Ampicillin (R) Azithromycin (S) Ceftazidime (R) Ciprofloxacin (R) Ceftriaxone (R) Imipenem (S)	Chloramphenicol (S) Gentamicin (R) Cefoxitin (R) Cefotaxime (R) Ampicillin (R) Azithromycin (S) Ceftazidime (R) Ciprofloxacin (R) Ceftriaxone (R) Imipenem (S)
Total Raw reads	4,323,964	5,184,842	5,014,788	6,401,456	4,502,008
Genome size (bp)	5,253,301	5,276,774	4,765,317	5,399,189	5,252,067
N50 value (bp)	192,656	194,064	94,357	192,557	192,679
No. Contigs	174	142	285	162	188
Coverage (×)	121.7	144.8	156	175.1	126.3
GC content (%)	50.5	50.5	50.5	50.5	50.5
Genome completeness	99.96	99.96	99.96	99.96	99.96
Genes (total)	5,224	5,223	5,091	5,426	5,223
CDSs (total)	5,142	5,129	5,000	5,332	5,138
Genes (coding)	4,930	4,943	4,718	5,132	4,931
CDSs (with protein)	4,930	4,943	4,718	5,132	4,931
Genes (RNA)	82	94	91	94	85
Multilocus sequence type (ST)	354	354	46	354	354
Core genome sequence type (cgST)	93,301	93,301	206,229	93,301	93,301
Phylogroup	F	F	A	F	F
Serotype	O153:H34	O153:H34	O9:H10	O153:H34	O153:H34
Plasmid replicon types	IncY_1, IncX1_1, IncFIB(pB171), IncFIA_1	IncY, IncFIB, IncFII, IncX, IncQ1, Col (MG828)	Col (MG828), IncFIB (pB171), IncFIA_1, IncX4_2, IncFIB (AP001918)	IncY_1, IncX1_1, IncFIB(pB171, Col8282_1	IncY_1, IncX1_1, IncFIB(pB171), IncFIA_1, Col8282_1
Antibiotic resistance genes	Beta-lactams (*bla*_CTX-M-15_, *bla_TEM_*_-1_), Fluoroquinolones (*qepA, gyrA_D87N, gyrA_S83L, parC_E84G, parC_S80I, parE_I355T*), Aminoglycosides (*aadA5, aph (6)-Id, aac (3)-IIe, aph(2'')-I, aph(3'')-Ib*), Sulfonamides (*sul2*), Tetracycline (*tetB***),** Fosfomycin (glpT_E448K, uhpT_E350Q), Trimethoprim (*dfrA17*)	Beta-lactams (*bla*_CTX-M-15_, *bla*_TEM-1_), Fluoroquinolones (*qepA, gyrA_D87N, gyrA_S83L, parC_E84G, parC_S80I, parE_I355T*), Aminoglycosides (*aadA5, aph(6)-Id, aac(3)-IIe, aph(2'')-I, aph(3'')-Ib*), Sulfonamides (*sul2*), Tetracycline (*tetB***),** Fosfomycin (glpT_E448K, uhpT_E350Q), Trimethoprim (*dfrA17*)	Beta-lactams (*bla*_TEM-1_), Fluoroquinolones (*qepA, gyrA_D87N, gyrA_S83L, parC_S57T, parC_S80I*), Macrolides (*mphA*), Aminoglycosides (*aph(6)-Id, aac (3)-IId, aph(3'')-Ib*), Sulfonamides (*sul2*), Tetracycline (*tetB*), Fosfomycin (glpT_E448K), Trimethoprim (*dfrA14*, *dfrA17*)	Beta-lactams (*bla*_CTX-M-15_, *bla*_TEM-1_), Fluoroquinolones (*qepA, gyrA_D87N, gyrA_S83L, parC_E84G, parC_S80I, parE_I355T*), Aminoglycosides (*aadA5, aph(6)-Id, aac(3)-IIe, aph(2'')-I, aph(3'')-Ib*), Sulfonamides (*sul2*), Tetracycline (*tetB*), Fosfomycin (glpT_E448K, uhpT_E350Q), Trimethoprim (*dfrA17*)	Beta-lactams (*bla*_CTX-M-15_, *bla*_TEM-1_), Fluoroquinolones (*qepA, gyrA_D87N, gyrA_S83L, parC_E84G, parC_S80I, parE_I355T*), Aminoglycosides (*aadA5, aph(6)-Id, aac(3)-IIe, aph(2'')-I, aph(3'')-Ib*), Sulfonamides (*sul2*), Tetracycline (*tetB*), Fosfomycin (glpT_E448K, uhpT_E350Q), Trimethoprim (*dfrA17*)
Accession number	JBPBLX000000000	JBPBLW000000000	JBPBLV000000000	JBPBLU000000000	JBPBLT000000000

**Fig 2:**
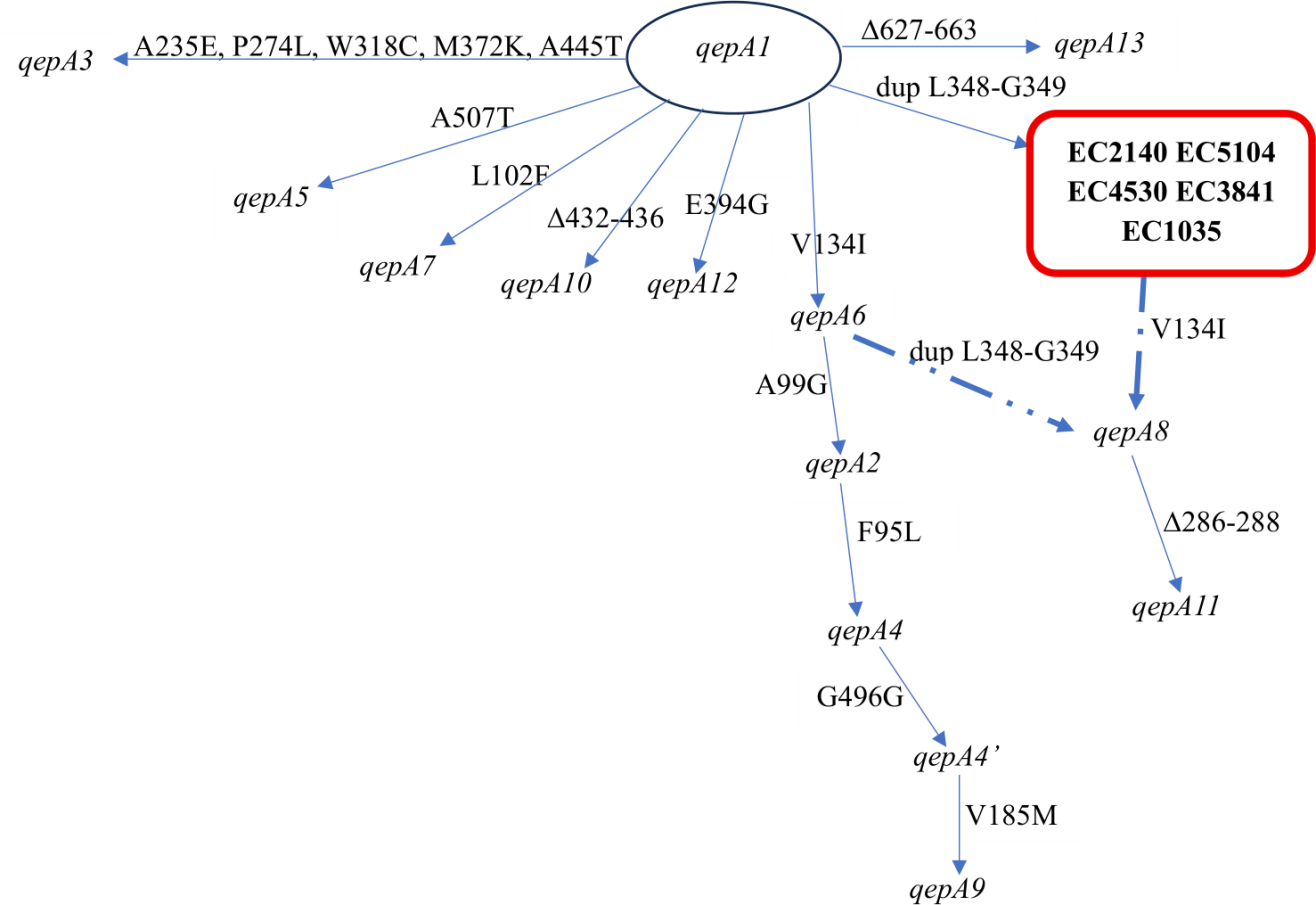
Evolutionary landscape of *qepA* variants. ∆286–288 *qepA11*, *qepA4*′ V185M *qepA9*: Mutational spectrum and distribution of *qepA* variants (*qepA*1–*qepA*13) identified across isolates. Specific amino acid substitutions (e.g., A235E, P274L, W318C, M372K, A445T), deletions (Δ286–288, Δ432–436), and duplications (L348–G349) are indicated. The EC strains are highlighted, showing the presence of distinct mutations within their genetic background. This schematic illustrates the diversification of *qepA* alleles and the accumulation of multiple mutations.

The *qepA* variant identified in this study shares 99.4% nucleotide identity with the reference sequence WP_202792279.1 and 99.81% amino acid identity with the reference sequence WP_109638576.1 but harbors unique amino acid substitutions distinct from previously reported alleles. Protein analysis of the isolates revealed that the *qepA* gene present had 513 amino acids with insertion of an L348–G349 duplication similar to *qepA8* but without a V134I substitution. The transmembrane topology prediction revealed that the *qepA* variant in the five isolates contained 14 transmembrane helices, arranged in 2 bundles of seven helices each, a characteristic feature of major facilitator superfamily (MFS) efflux pumps consistent with the structural organization of MFS transporters. This topology supports the putative role of *qepA* in mediating active efflux of fluoroquinolones across the bacterial inner membrane through an alternating access mechanism. The L348–G349 amino acid duplication was located within the periplasmic loop connecting TM9 and TM10, which could suggest a possible influence on loop flexibility or substrate passage dynamics representing evolutionary divergence among *qepA* variants as depicted in Fig. 2. Structural modeling and superimposition showed minimal deviation between the *qepA*1 and *qep*A8 and variant proteins (RMSD = 0.64–0.75 Å across aligned core residues), indicating that the L348–G349 duplication does not significantly alter the overall structure or predicted topology of *qepA*. A limitation of this study is the absence of phenotypic and functional characterization of the novel *qepA* variant. Further investigation is needed to determine the functional significance of this unique *qepA* allele type and its contribution to high ciprofloxacin MIC levels.

## Supplementary Material

Reviewer comments

## Data Availability

This whole-genome shotgun project has been deposited at GenBank under accession number PRJNA1192017. Genome accession numbers for the assembled reads are JBPBLT000000000–JBPBLX000000000. The sequence of the qepA variant being described in all five isolates has been deposited as part of the whole-genome sequencing data in NCBI.

## SUPPLEMENTAL MATERIAL

OPEN PEER REVIEW reviewer-comments.pdf An accounting of the reviewer comments and feedback.

ASM does not own the copyrights to Supplemental Material that may be linked to, or accessed through, an article. The authors have granted ASM a non-exclusive, world-wide license to publish the Supplemental Material files. Please contact the corresponding author directly for reuse.
