# Supplementary material for: Fluoroquinolone-resistant Escherichia coli carrying quinolone efflux pump (qepA) with L348–G349 duplication in the absence of V134I substitution
Source: Microbiol Spectr. 2026 Jun 29;14(8):e00056-26. doi: 10.1128/spectrum.00056-26 (PMC13435699; doi:10.1128/spectrum.00056-26)
Supplement: Supplementary file 1 [file reviewer-comments.pdf]

AMERICAN SOCIETY FOR MICROBIOLOGY | Microbiology Spectrum

Fluoroquinolone-resistant *Escherichia coli* carrying *quinolone efflux pump* (*qepA*) with L348-G349 duplication in the absence of V134I substitution.

Kevin Kariuki, Kirkby D Tickell, Collins Kigen, James Wachira, Polycarp Mogeni, Doreen Rwigi, Cecilia Mbae, Kelvin Kering, Benson Singa, Judd Walson, Samuel Kariuki, Patricia B. Pavlinac, and Ferric Fang

Corresponding Author(s): Kevin Kariuki, Kenya Medical Research Institute

Review Timeline:

Submission Date:	January 9, 2026
Editorial Decision:	February 27, 2026
Revision Received:	May 8, 2026
Editorial Decision:	May 17, 2026
Revision Received:	May 20, 2026
Accepted:	May 27, 2026

Editor: Philips Akinwole

Reviewer(s): Disclosure of reviewer identity is with reference to reviewer comments included in decision letter(s). The following individuals involved in review of your submission have agreed to reveal their identity: Sébastien Bontemps-Gallo (Reviewer #2)

Transaction Report:

(Note: With the exception of the correction of typographical or spelling errors that could be a source of ambiguity, letters and reports are not edited. The original formatting of letters and referee reports may not be reflected in this compilation.)

DOI: https://doi.org/10.1128/spectrum.00056-26

Re: Spectrum00056-26 (**Fluoroquinolone-resistant *Escherichia coli* carrying *quinolone efflux pump* (*qepA*) with L348-G349 duplication in the absence of V134I substitution**)

Dear Mr. Kevin Kariuki:

Thank you for the privilege of reviewing your work. Below you will find my comments, instructions from the Spectrum editorial office, and the reviewer comments at the end of the email.

The authors identified a qepA variant with an L348-G349 amino acid duplication, similar to qepA8 but lacking the V134I substitution, and characterized its genomic context, predicted transmembrane topology, and associated resistance determinants.

Here are my comments to improve the quality of this study.
1) The isolates harbor both multiple QRDR mutations and the novel qepA variant, yet the manuscript sometimes implies that this allele may "contribute to high-level resistance" (lines 36, 51). These statements risk over-attributing the phenotype to the efflux pump without functional testing or comparative genomics against qepA-negative but otherwise similar isolates. PMQR (including qepA) typically confers low-level resistance and shifts mutant selection windows.
(Jacoby, G. A., Strahilevitz, J., & Hooper, D. C. (2015). Plasmid-mediated quinolone resistance. Plasmids: Biology and Impact in Biotechnology and Discovery, 475-503.
Martínez-Martínez, L., Eliecer Cano, M., Manuel Rodríguez-Martínez, J., Calvo, J., & Pascual, A. (2008). Plasmid-mediated quinolone resistance. Expert review of anti-infective therapy, 6(5), 685-711.)

2) The study analyzes five isolates from a specific subset of children (hospitalized, non-traumatic admissions, discharged within a trial). The manuscript sometimes generalizes to "clinical settings in sub-Saharan Africa" or implies broader circulation, without showing prevalence data. Also, with no denominator for how many isolates were screened for qepA or how these five were selected. Surveillance/generalizability claims should reflect the narrow sample and opportunistic nature of isolate selection. Rephrase broader statements to emphasize that these isolates illustrate ongoing diversification rather than demonstrate prevalence or regional spread/ avoid population-level inference.

3) Methods: Briefly specify criteria for calling a sequence a novel qepA allele (e.g., ">X% identity to known alleles but with unique ...).

4) Data availability: Add a short sentence indicating whether the qepA allele sequence and its annotation have been submitted separately to a resistance gene database (e.g., CARD or ResFinder).

5) Revise Figure 1:Fig. 1 (genetic context) is informative, but the legend could be more explicit about the scale, orientation, and sequence identity between the two shown strains.
Add scale bar (bp) and explicitly mark the boundaries of qepA, intI1, and ISCR3.
Include a short note on sequence identity between the two contexts or any structural differences.

After abbreviating minimum inhibitory concentrations (MICs) at first mention, MIC should be used subsequently (see lines 35, 62, 74).

Please return the manuscript within 60 days; if you cannot complete the modification within this time period, please contact me. If you do not wish to modify the manuscript and prefer to submit it to another journal, notify me immediately so that the manuscript may be formally withdrawn from consideration by Spectrum.

Revision Guidelines
To submit your modified manuscript, log into the submission site at https://spectrum.msubmit.net/cgi-bin/main.plex. Go to Author Tasks and click the appropriate manuscript title to begin. The information you entered when you first submitted the paper will be displayed; update this as necessary. Note the following requirements:

• Upload point-by-point responses to the issues raised by the reviewers in a file named "Response to Reviewers," NOT in your cover letter.
• Upload a compare copy of the manuscript (without figures) as a "Marked-Up Manuscript" file.
• Upload a clean .DOC/.DOCX version of the revised manuscript and remove the previous version.
• Each figure must be uploaded as a separate, editable, high-resolution file (TIFF or EPS preferred), and any multipanel figures must be assembled into one file.
• Any supplemental material intended for posting by ASM should be uploaded with their legends separate from the main manuscript. You can combine all supplemental material into one file (preferred) or split it into a maximum of 10 files with all associated legends included.

For complete guidelines on revision requirements, see our Submission and Review Process webpage. Submission of a paper

that does not conform to guidelines may delay acceptance of your manuscript.

Data availability: ASM policy requires that data be available to the public upon online posting of the article, so please verify all links to sequence records, if present, and make sure that each number retrieves the full record of the data. If a new accession number is not linked or a link is broken, provide Spectrum production staff with the correct URL for the record. If the accession numbers for new data are not publicly accessible before the expected online posting of the article, publication may be delayed; please contact production staff (Spectrum@asmusa.org) immediately with the expected release date.

Publication Fees: For information on publication fees and which article types are subject to charges, visit our website. If your manuscript is accepted for publication and any fees apply, you will be contacted separately about payment during the production process; please follow the instructions in that e-mail. Arrangements for payment must be made before your article is published.

ASM Membership: Corresponding authors may join or renew ASM membership to obtain discounts on publication fees. Need to upgrade your membership level? Please contact Customer Service at Service@asmusa.org.

The ASM Journals program strives for constant improvement in our submission and publication process. Please tell us how we can improve your experience by taking this quick Author Survey.

Thank you for submitting your paper to Spectrum.

Sincerely,
Philips Akinwole
Editor
Microbiology Spectrum

Reviewer #2 (Comments for the Author):

This manuscript by Kariuki and collaborators reports the identification of a novel qepA variant in fluoroquinolone-resistant Escherichia coli isolates from Kenyan children under five. The variant features a unique L348-G349 duplication (without the V134I substitution typical of qepA8). The work highlights the evolutionary diversification of plasmid-mediated resistance in sub-Saharan Africa, with implications for antimicrobial stewardship.

1/ While this study reports this mutation for the first time, it would be interesting to know how frequently this mutation has been identified. The manuscript indicates that the isolates were obtained during the Toto Bora trial, which included 1,400 children. Among these individuals, what percentage carried the mutation? In other words, are the five isolated cases unique among the 1,400 participants?

2/ Why focus on children under five? Is there evidence of higher carriage rates or unique resistance mechanisms in this age group?

3/ What is the prevalence of fluoroquinolone use in the studied hospitals ?

4/ The qepA variant is located on a plasmid. What is the risk of horizontal transfer to other pathogens?

5/ How does the MIC of this variant compare to qepA8 or other alleles?

6/ Did the authors perform a 3D structural modeling (AlphaFold) analysis to assess any structural impact of the L348-G349 duplication mutation?

RE: **Fluoroquinolone-resistant *Escherichia coli* carrying *quinolone efflux pump* (*qepA*) with L348-G349 duplication in the absence of V134I substitution.** (Spectrum00056-26)

Reviewer #1 (Comments for the Author):

Here are my comments to improve the quality of this study.

1) The isolates harbor both multiple QRDR mutations and the novel qepA variant, yet the manuscript sometimes implies that this allele may "contribute to high-level resistance" (lines 36, 51). These statements risk over-attributing the phenotype to the efflux pump without functional testing or comparative genomics against qepA-negative but otherwise similar isolates. PMQR (including *qepA*) typically confers low-level resistance and shifts mutant selection windows.

(Jacoby, G. A., Strahilevitz, J., & Hooper, D. C., 2015). Plasmid-mediated quinolone resistance. Plasmids: Biology and Impact in Biotechnology and Discovery, 475-503.

Martínez-Martínez, L., Eliecer Cano, M., Manuel Rodríguez-Martínez, J., Calvo, J., & Pascual, A. (2008). Plasmid-mediated quinolone resistance. Expert review of anti-infective therapy, 6(5), 685-711.)

Response: We thank the reviewer for this important comment. We agree that PMQR determinants such as *qepA* are typically associated with low-level resistance and may primarily act to expand the mutant selection window to favour subsequent development of high-level resistance rather than directly conferring high-level resistance. Our isolates harbour multiple QRDR mutations, and we acknowledge that these chromosomal mutations are directly responsible for the high-level resistance phenotype. We have revised the manuscript on lines 36 and 51 to clarify that the contribution of the unique *qepA* variant should be interpreted as a likely accessory role. We also note that functional validation would be required to definitively determine its phenotypic impact of the unique *qepA* variant.

2) The study analyzes five isolates from a specific subset of children (hospitalized, non-traumatic admissions, discharged within a trial). The manuscript sometimes generalizes to "clinical settings in sub-Saharan Africa" or implies broader circulation, without showing prevalence data. Also, with no denominator for how many isolates were screened for qepA or how these five were selected. Surveillance/generalizability claims should reflect the narrow sample and opportunistic nature of isolate selection. Rephrase broader statements to emphasize that these isolates illustrate ongoing diversification rather than demonstrate prevalence or regional spread/ avoid population-level inference.

Response: We thank the reviewer for this comment and agree that the scope of inference should reflect the study design and sampling framework. To address this, we have clarified the denominator and selection process in the revised manuscript in lines 68-71. Specifically, a total

of 188 fluoroquinolone non-susceptible *E. coli* isolates were screened for *qepA*, of which 16 (9%) were positive. The five isolates included in this study were selected from this subset for further characterization based on whole-genome sequencing and variant analysis.

We have revised the text throughout to avoid overgeneralization and to more accurately reflect the opportunistic and cohort-specific nature of the sampling (i.e., children enrolled in a defined clinical trial). Statements referring to broader circulation in sub-Saharan Africa have been tempered to indicate that our findings are hypothesis-generating and consistent with, rather than representative of, regional trends.

3) Methods: Briefly specify criteria for calling a sequence a novel qepA allele (e.g., ">X% identity to known alleles but with unique ...).

Response: We thank the reviewer for this suggestion. We have revised the Methods section to explicitly define the criteria used to classify a *qepA* sequence as a **unique** allele in lines 98-103. Briefly, to identify the amino acid duplication, other alleles of the *qepA* reference sequences were downloaded from NCBI and aligned in MEGA. Additionally, the *qepA* sequences were compared against reference alleles in CARD using nucleotide and amino acid sequences. Sequence identity was based on the overall similarity to known *qepA* alleles (>99% nucleotide identity), while uniqueness was based on distinct nucleotide or amino acid mutation combinations not reported in curated databases or other publications.

The *qepA* variant identified in this study shares 99.4% nucleotide identity with the reference sequence WP_202792279.1 but harbors a unique amino acid mutation combination that distinguishes it from previously reported alleles. We acknowledge that formal designation of a new allele would require submission to and curation by a reference database (e.g., CARD), and we have clarified this as a unique allele in the revised manuscript.

4) Data availability: Add a short sentence indicating whether the qepA allele sequence and its annotation have been submitted separately to a resistance gene database (e.g., CARD or ResFinder).

Response: We thank the reviewer for this comment. We have added a statement in the Data Availability section to clarify the status of the *qepA* allele. The sequence of the *qepA* variant being described in all five isolates has been deposited as part of the whole-genome sequencing data in NCBI. In addition, submission to a curated resistance gene database, CARD (https://github.com/arpcard/amr_curation/issues/127), is in progress, and a submission to ResFinder has been made for review.

5) Revise Figure 1: Fig. 1 (genetic context) is informative, but the legend could be more explicit

about the scale, orientation, and sequence identity between the two shown strains. Add scale bar (bp) and explicitly mark the boundaries of *qepA*, intI1, and ISCR3. Include a short note on sequence identity between the two contexts or any structural differences.

Response: We thank the reviewer for this constructive suggestion. We have revised Figure 1 and its legend to improve clarity and interpretability. Specifically, we have added a scale bar (1 kb) to indicate genomic distance and ensured consistent left-to-right orientation of all contigs to facilitate direct comparison. The boundaries of key genetic elements, including *qepA*, *intI1*, and *ISCR3*, are now explicitly indicated and clearly labelled within the figure. We have also included a statement in the figure legend describing the high level of sequence conservation across the *qepA*-containing regions, as indicated by the alignment shading.

6) After abbreviating minimum inhibitory concentrations (MICs) at first mention, MIC should be used subsequently (see lines 35, 62, 74).

Response: We thank the reviewer for noting this. The abbreviations have been included accordingly after the first mention and abbreviated thereafter.

Reviewer #2 (Comments for the Author):

This manuscript by Kariuki and collaborators reports the identification of a novel qepA variant in fluoroquinolone-resistant Escherichia coli isolates from Kenyan children under five. The variant features a unique L348-G349 duplication (without the V134I substitution typical of qepA8). The work highlights the evolutionary diversification of plasmid-mediated resistance in sub-Saharan Africa, with implications for antimicrobial stewardship.

1/ While this study reports this mutation for the first time, it would be interesting to know how frequently this mutation has been identified. The manuscript indicates that the isolates were obtained during the Toto Bora trial, which included 1,400 children. Among these individuals, what percentage carried the mutation? In other words, are the five isolated cases unique among the 1,400 participants?

Response: We thank the reviewer for this important observation and agree that the scope of inference should reflect the study design and sampling framework. To address this, we have clarified the denominator and selection process in the revised manuscript in lines 68-71. Specifically, a total of 188 fluoroquinolone non-susceptible *E. coli* isolates were screened for *qepA*, of which 16 (9%) were positive. The five isolates included in this study were selected from this subset for further characterization based on whole-genome sequencing and variant analysis.

2/ Why focus on children under five? Is there evidence of higher carriage rates or unique resistance mechanisms in this age group?

Response: We appreciate the reviewer's important question regarding the focus on children under five years of age. This population was selected because young children are a well-recognized high-risk group for intestinal carriage and colonization of antimicrobial-resistant Enterobacterales. Several factors contribute to this, including frequent exposure to antibiotics, longer hospital stays, and environmental exposures in limited-resource settings. This makes them an important reservoir for the acquisition and transmission of resistance genes, including plasmid-mediated mechanisms such as *qepA*.

From a study design perspective, the Toto Bora trial specifically enrolled children under five years following hospital discharge, a period associated with increased vulnerability to infection and antibiotic exposure. This cohort, therefore, provides a relevant framework for investigating the emergence and dissemination of resistance mechanisms in a high-risk population.

While our study was not designed to compare resistance mechanisms across age groups, focusing on this population allows us to capture clinically and epidemiologically significant resistance patterns at an early stage, which may have implications for both treatment and transmission dynamics.

3/ What is the prevalence of fluoroquinolone use in the studied hospitals?

Response: We thank the reviewer for this important question. Fluoroquinolone use in the study population was low, with inpatient administration documented in 0.9% of participants during hospitalization. Among children with an *E. coli* isolate carrying the *qepA* gene, none of them was among those administered ciprofloxacin during their hospital stays.

4/ The qepA variant is located on a plasmid. What is the risk of horizontal transfer to other pathogens?

Response: We thank the reviewer for this important question regarding the potential for horizontal transfer. No relaxase, mating pair formation (MPF) system, or origin of transfer (*oriT*) genes were detected in the *qepA*-carrying plasmids in all five isolates, indicating that the plasmids are non-mobilizable. However, as described in Figure 1, the genetic context of *qepA* finds the gene to be flanked by intI1 and ISCR3, suggesting that the resistance determinant is associated with a mobile genetic element. This may enable horizontal dissemination of *qepA* at the gene or composite transposon level, even in the absence of autonomous plasmid transfer machinery. The presence of integron- and ISCR-associated mobile elements suggests a risk of horizontal gene

mobilization within Enterobacterales populations, particularly in environments where multiple mobile genetic elements coexist.

5/ How does the MIC of this variant compare to qepA8 or other alleles?

Response: We thank the reviewer for this question. In our dataset, phenotypic susceptibility testing was available for isolates carrying *qepA1*, *qepA4*, and the variant described in this study. The MICs for ciprofloxacin were consistently high (≥32 µg/mL) across these isolates, with no observable difference in resistance levels between the variant and the *qepA1* or *qepA4* carrying *E. coli* isolates. It is likely that this high-level resistance resulted from concomitant QRDR mutations rather than from the *qepA* loci per se. We do not have isolates carrying *qepA8* or other *qepA* alleles within our collection, therefore a direct comparative assessment against those variants was not possible.

6/ Did the authors perform a 3D structural modelling (AlphaFold) analysis to assess any structural impact of the L348-G349 duplication mutation?

Response: We thank the reviewer for this suggestion. The reference sequence qepA1 (WP 012372821.1) and the query sequence (EC1035) were loaded on ColabFold v1.6.1: AlphaFold2 using MMseqs2 and superimposed on UCSF ChimeraX v1.11.1. A statement on the results has been added in the manuscript, which now reads "Structural modelling and superimposition showed minimal deviation between the *qepA*1 and *qep*A8 and variant proteins (RMSD = 0.64–0.75 Å across aligned core residues), indicating that the L348–G349 duplication does not significantly alter the overall structure or predicted topology of *qepA*." The higher RMSD observed when including all residues reflects variability in flexible regions rather than global structural changes.

Reviewer #3 (Comments for the Author):

Major concerns

1. Epidemiological relatedness of the isolates

The five isolates originate from two different hospitals, but the manuscript does not specify which isolate comes from which institution. This information is essential for the epidemiological interpretation.

Response: We thank the reviewer for highlighting the importance of clarifying the epidemiological relatedness of these isolates. We have now explicitly indicated the hospital (county) of origin for each isolate in the revised table (Table 1), as suggested. Regarding clonality, four of the five isolates share the same serotype and sequence type (ST). However, these isolates

were recovered from children discharged from different hospitals, suggesting that they are unlikely to represent a single hospital-based outbreak or point-source transmission event.

Furthermore, four of the five isolates share the same serotype and sequence type. This raises the important question of whether these isolates are clonal. If these four isolates also originate from the same hospital, it is highly likely that they represent a single strain rather than independent occurrences. The authors should:
o clearly indicate the hospital of origin for each isolate, - Indicated
o provide an analysis of clonality (e.g., cgMLST/SNP-based comparison or at least a detailed discussion based on the available typing data).

Response: We performed cgMLST-based hierarchical clustering (HierCC) on Enterobase v1.2.0 to assess the relatedness of the isolates. While four of the five isolates shared the same sequence type, they were assigned to distinct HC0 clusters, indicating that they are not genetically identical and do not represent a single clonal strain. At HC20–HC50, these isolates were grouped within the same cluster, consistent with their belonging to a common lineage.

A sentence in the results section has been added in this regard, L115-117: "cgMLST analysis revealed that four isolates belonged to the same core genome sequence type (cgST 93301), while EC4530 belonged to a distinct cgST (206229). Despite sharing the same MLST, the isolates were assigned to distinct HC0 clusters, excluding clonality, while clustering at higher HierCC levels (HC20–HC50), indicating a common lineage without evidence of recent transmission."

2. Lack of phenotypic characterization of the novel qepA variant
The description of the new qepA variant is currently limited to sequence analysis. No phenotypic data are presented to demonstrate:
o the contribution of this variant to quinolone resistance compared with previously described qepA variants, and
o its effect in a genetic background lacking gyrA and parC mutations.
Without such data, the functional relevance of the novel variant remains unclear. At a minimum, the authors should provide susceptibility data from a suitable transformant or transconjugant carrying the plasmid in a quinolone-susceptible recipient.

Response: We thank the reviewer for this important comment. We agree that functional characterization using a controlled genetic background would provide more definitive evidence of the contribution of the unique *qepA* variant to quinolone resistance. In the present study, our analysis was limited to sequence-based characterization and phenotypic susceptibility testing of the clinical isolates, in which ciprofloxacin MICs were consistently elevated (≥32 µg/mL). However, these isolates also harbor multiple chromosomal mutations in *gyrA* and *parC* determinants, which

precludes direct attribution of the resistance phenotype to the *qepA* variant alone. We acknowledge that transformation or conjugation of the *qepA*-carrying plasmid into a quinolone-susceptible recipient strain, followed by comparative susceptibility testing, would allow for a more precise assessment of the functional impact of this variant relative to previously described *qepA* alleles. This experiment was beyond the scope of the current study but represents an important direction for future work.

3. Plasmid analysis

A comparative analysis of the plasmid regions surrounding the new qepA variant in all five isolates is missing. This is important to determine whether:

o the gene is located on the same plasmid backbone in all isolates, and o a common mobile genetic element is involved. In addition, the plasmid incompatibility (Inc) type(s) carrying the new qepA variant should be identified and reported.

Response: We thank the reviewer for this important suggestion. Plasmid replicon typing was performed using PlasmidFinder, which identified multiple incompatibility (Inc) group(s) associated with *qepA*-positive isolates. Our analysis shows that the *qepA* gene is consistently associated with a conserved genetic structure comprising *intI1* and IS91-like elements (e.g., *ISCR3*), suggesting a common mobile genetic platform. Plasmid reconstruction using Mobsuite revealed that the *qepA* gene is located on a non-mobilizable plasmid with no relaxase, mating pair formation (MPF) system, or origin of transfer (*oriT*) genes across all five *qepA*-carrying isolates. We have now included these findings in the Results section and report the identified plasmid replicon types. Due to the use of short-read sequencing data, complete plasmid reconstruction was not possible, and therefore, definitive assignment of *qepA* to specific plasmids could not be done.

4. Horizontal transferability

No conjugation experiments are presented. Demonstrating whether the plasmid(s) harboring the novel qepA variant are transferable is crucial for assessing their epidemiological and clinical relevance.

Response: We thank the reviewer for this important point. We agree that experimental demonstration of plasmid transferability would provide valuable insight into the epidemiological and clinical relevance of the *qepA*-carrying plasmids. In this study, conjugation assays were not performed, and therefore, we cannot directly assess transferability under laboratory conditions.

However, in silico analysis of the plasmid sequences showed no identifiable relaxase genes, mating pair formation (MPF) systems, or origin of transfer (*oriT*) sequences necessary for

conjugative transfer, suggesting that the plasmids are likely non-conjugative. Notably, the *qepA* gene was located within a genetic context flanked by **intI1** and **ISCR3**, indicating that it resides in a mobile resistance region capable of mobilization via recombination or in trans with the help of co-resident conjugative elements. Therefore, we interpret the horizontal transfer potential as likely limited at the plasmid level but not negligible at the gene level. We acknowledge that conjugation or mobilization assays using suitable recipient strains would be required to experimentally validate transferability, and this represents an important direction for future work.

Specific comment

• Line 35: The ciprofloxacin resistance reported for the isolates is most likely primarily due to the mutations in *gyrA* and *parC*, rather than the presence of *qepA*. This should be clearly stated and discussed to avoid overinterpretation of the role of the plasmid-mediated resistance determinant.

Response: We thank the reviewer for this important clarification. We agree that chromosomal mutations in *gyrA* and *parC* are the primary drivers of high-level ciprofloxacin resistance in our isolates. We have revised the manuscript to highlight the accessory role of *qepA* as a contributing factor consistent with PMQR determinants, which typically confer low-level resistance but may facilitate the selection of higher-level resistance. These revisions ensure that the interpretation of the resistance phenotype is appropriately balanced and avoids overattribution to the plasmid-mediated mechanism. The statement now reads L36-39 "The evolution of *qepA* indicates the persistence and diversification of transferable quinolone resistance determinants conferring low-level resistance, which may be facilitating further selection and dissemination of high-level fluoroquinolone resistance, expanding the mutant selection window in *E. coli* strains found in sub-Saharan Africa (SSA)".

The identification of a new qepA variant is potentially interesting, but the manuscript currently lacks essential epidemiological, plasmid, and functional data. Addressing the points above would substantially strengthen the study and allow a more meaningful assessment of the clinical and microbiological significance of the finding.

Response: We have now provided additional information regarding epidemiological relatedness, plasmid content and the functional significance of *qepA*. We hope that the reviewer finds the revised manuscript to be suitable for publication.

Re: Spectrum00056-26R1 (**Fluoroquinolone-resistant *Escherichia coli* carrying *quinolone efflux pump* (*qepA*) with L348-G349 duplication in the absence of V134I substitution**)

Dear Mr. Kevin Kariuki:

Thank you for the privilege of reviewing your work. Below you will find my comments, instructions from the Spectrum editorial office, and the reviewer comments.

I think that the revised version of the manuscript addresses the reviewers' concerns, and the conclusions are now well supported. I only have two minor suggestions:

Figure 1: Some annotations are currently difficult to read because they do not contrast sufficiently with the gray background.
Response to Reviewer 3, Comment 2: Regarding the lack of phenotypic characterization of the novel qepA variant, the authors note that functional characterization is beyond the scope of the current study. However, this should be explicitly stated as a limitation in the manuscript.

We invite you to revise your paper with careful attention to these suggestions. Once the revision is complete, please submit the updated manuscript along with a point-by-point response to the reviewers. This will help us move toward a swift decision.

Please return the manuscript within 60 days; if you cannot complete the modification within this time period, please contact me. If you do not wish to modify the manuscript and prefer to submit it to another journal, notify me immediately so that the manuscript may be formally withdrawn from consideration by Spectrum.

Revision Guidelines
To submit your modified manuscript, log into the submission site at https://spectrum.msubmit.net/cgi-bin/main.plex. Go to Author Tasks and click the appropriate manuscript title to begin. The information you entered when you first submitted the paper will be displayed; update this as necessary. Note the following requirements:

• Upload point-by-point responses to the issues raised by the reviewers in a file named "Response to Reviewers," NOT in your cover letter.
• Upload a compare copy of the manuscript (without figures) as a "Marked-Up Manuscript" file.
• Upload a clean .DOC/.DOCX version of the revised manuscript and remove the previous version.
• Each figure must be uploaded as a separate, editable, high-resolution file (TIFF or EPS preferred), and any multipanel figures must be assembled into one file.
• Any supplemental material intended for posting by ASM should be uploaded with their legends separate from the main manuscript. You can combine all supplemental material into one file (preferred) or split it into a maximum of 10 files with all associated legends included.

For complete guidelines on revision requirements, see our Submission and Review Process webpage. Submission of a paper that does not conform to guidelines may delay acceptance of your manuscript.

Data availability: ASM policy requires that data be available to the public upon online posting of the article, so please verify all links to sequence records, if present, and make sure that each number retrieves the full record of the data. If a new accession number is not linked or a link is broken, provide Spectrum production staff with the correct URL for the record. If the accession numbers for new data are not publicly accessible before the expected online posting of the article, publication may be delayed; please contact production staff (Spectrum@asmusa.org) immediately with the expected release date.

Publication Fees: For information on publication fees and which article types are subject to charges, visit our website. If your manuscript is accepted for publication and any fees apply, you will be contacted separately about payment during the production process; please follow the instructions in that e-mail. Arrangements for payment must be made before your article is published.

ASM Membership: Corresponding authors may join or renew ASM membership to obtain discounts on publication fees. Need to upgrade your membership level? Please contact Customer Service at Service@asmusa.org.

The ASM Journals program strives for constant improvement in our submission and publication process. Please tell us how we can improve your experience by taking this quick Author Survey.

Thank you for submitting your paper to Spectrum.

Sincerely,
Philips Akinwole

Editor
Microbiology Spectrum

RE: **Fluoroquinolone-resistant *Escherichia coli* carrying *quinolone efflux pump* (*qepA*) with L348-G349 duplication in the absence of V134I substitution.** (Spectrum00056-26)

Editorial comments

Thank you for the privilege of reviewing your work. Below you will find my comments, instructions from the Spectrum editorial office, and the reviewer comments.

I think that the revised version of the manuscript addresses the reviewers' concerns, and the conclusions are now well supported. I only have two minor suggestions:

Response: We sincerely thank the editor and the reviewers for the thorough review of this work. We have now addressed the editorial comments. We hope that the editor and reviewers will find the revised manuscript to be suitable for publication.

Comment 1#

Figure 1: Some annotations are currently difficult to read because they do not contrast sufficiently with the grey background.

Response: We thank the reviewer for this observation. We have revised Figure 1 to improve readability by increasing the contrast between annotations and the background shading. Specifically, annotation colours and font formatting were adjusted to enhance visibility and ensure that all gene labels and feature annotations are clearly distinguishable in both digital and print formats.

Comment 2#

Response to Reviewer 3, Comment 2: Regarding the lack of phenotypic characterization of the novel qepA variant, the authors note that functional characterization is beyond the scope of the current study. However, this should be explicitly stated as a limitation in the manuscript.

Response: We appreciated this comment and have added the following comment on L144 and 145: “A limitation of this study is the absence of phenotypic and functional characterization of the novel *qepA* variant.”

Re: Spectrum00056-26R2 (**Fluoroquinolone-resistant *Escherichia coli* carrying *quinolone efflux pump* (*qepA*) with L348-G349 duplication in the absence of V134I substitution.**)

Dear Mr. Kevin Kariuki:

Your manuscript has been accepted, and I am forwarding it to the ASM production staff for publication. Your paper will first be checked to make sure all elements meet the technical requirements. ASM staff will contact you if anything needs to be revised before copyediting and production can begin. Otherwise, you will be notified when your proofs are ready to be viewed.

Data Availability: ASM policy requires that data be available to the public upon online posting of the article, so please verify all links to sequence records, if present, and make sure that each number retrieves the full record of the data. If a new accession number is not linked or a link is broken, provide production staff with the correct URL for the record. If the accession numbers for new data are not publicly accessible before the expected online posting of the article, publication may be delayed; please contact ASM production staff immediately with the expected release date.

Publication Fees: For information on publication fees and which article types have charges, please visit our website. We have partnered with Copyright Clearance Center (CCC) to collect author charges. If fees apply to your paper, you will receive a message from no-reply@copyright.com with further instructions. For questions related to paying charges through RightsLink, please contact CCC at ASM_Support@copyright.com or toll free at +1-877-622-5543. CCC makes every attempt to respond to all emails within 24 hours.

ASM Membership: Corresponding authors may join or renew ASM membership to obtain discounts on publication fees. Need to upgrade your membership level? Please contact Customer Service at Service@asmusa.org.

Embargo Policy: A press release may be issued as soon as the manuscript is posted on the Spectrum Latest Articles webpage. The corresponding author will receive an email with the subject line "ASM Journals Author Services Notification" when the article is available online.

The ASM Journals program strives for constant improvement in our submission and publication process. Please tell us how we can improve your experience by taking this quick Author Survey.

Thank you for submitting your paper to Spectrum.

Sincerely,
Philips Akinwole
Editor
Microbiology Spectrum
